# Shock waves generated by toroidal bubble collapse are imperative for kidney stone dusting during Holmium:YAG laser lithotripsy

**DOI:** 10.1016/j.ultsonch.2023.106649

**Published:** 2023-10-15

**Authors:** Gaoming Xiang, Junqin Chen, Derek Ho, Georgy Sankin, Xuning Zhao, Yangyuanchen Liu, Kevin Wang, John Dolbow, Junjie Yao, Pei Zhong

**Affiliations:** a Thomas Lord Dept. of Mechanical Engineering and Materials Science, Duke University, Durham, NC 27708, USA; b Dept. of Biomedical Engineering, Duke University, Durham, NC 27708, USA; c Dept. of Aerospace and Ocean Engineering, Virginia Polytechnic Institute and State University, Blacksburg, VA 24061, USA; 1 Current address: Optics and Thermal Radiation Research Center, Institute of Frontier and Interdisciplinary Science, Shandong University, Qingdao 266237, China

## Abstract

Holmium:yttrium–aluminum-garnet (Ho:YAG) laser lithotripsy (LL) has been the treatment of choice for kidney stone disease for more than two decades, yet the mechanisms of action are not completely clear. Besides photothermal ablation, recent evidence suggests that cavitation bubble collapse is pivotal in kidney stone dusting when the Ho:YAG laser operates at low pulse energy (*E_p_*) and high frequency (*F*). In this work, we perform a comprehensive series of experiments and model-based simulations to dissect the complex physical processes in LL. Under clinically relevant dusting settings (*E_p_* = 0.2 J, *F* = 20 Hz), our results suggest that majority of the irradiated laser energy (>90 %) is dissipated by heat generation in the fluid surrounding the fiber tip and the irradiated stone surface, while only about 1 % may be consumed for photothermal ablation, and less than 0.7 % is converted into the potential energy at the maximum bubble expansion. We reveal that photothermal ablation is confined locally to the laser irradiation spot, whereas cavitation erosion is most pronounced at a fiber tip-stone surface distance about 0.5 mm where multi foci ring-like damage outside the thermal ablation zone is observed. The cavitation erosion is caused by the progressively intensified collapse of jet-induced toroidal bubble near the stone surface (<100 μm), as a result of Raleigh-Taylor and Richtmyer-Meshkov instabilities. The ensuing shock wave-stone interaction and resultant leaky Rayleigh waves on the stone surface may lead to dynamic fatigue and superficial material removal under repeated bombardments of toroidal bubble collapses during dusting procedures in LL.

## Highlights

• Shock waves from toroidal bubble collapse is imperative for kidney stone dusting.
• Progressively intensified collapse of toroidal bubbles boosts shock wave emission.
• The shock waves, not jet impact, are vital for cavitation damage in laser lithotripsy.
• The leaky Rayleigh waves may contribute to superficial material removal.

## 1 Introduction

Lasers have been used in a wide range of medical applications in urology [1], ophthalmology [2], dermatology [3], biomedical imaging [4], drug delivery [5], and tumor therapy [6]. Over the past two decades, Holmium:yttrium–aluminum-garnet (Ho:YAG) laser lithotripsy (LL) has been the gold standard in the surgical management of kidney stone disease (KSD) [1], [7], [8], [9], which is associated with healthcare expenditures over $2 billion/year in the US [10]. Compared to other treatment options for KSD, such as shock wave lithotripsy (SWL), LL via ureteroscopy is effective for renal calculi of all chemical compositions, leading to better stone clearance and lower recurrence rate [11].

Recent advances in laser technologies have significantly altered the prevailing clinical strategy of LL from fragmenting using high pulse energy (*E_p_* = 0.8–1.2 J) and low pulse repetition frequency (PRF = 1–10 Hz) to dusting at low *E_p_* (=0.2–0.4 J) and high PRF (=12–100 Hz) [8], [12], [13], [14], [15]. During LL using Ho:YAG laser (λ = 2080 nm, optical penetration depth in water = 0.4 mm, pulse duration > 70 μs), an elongated vapor bubble is typically formed due to superheating of the fluid at the fiber tip [16], which facilitates the laser energy transmission to the kidney stone through the Moses effect [17]. As a result, photothermal ablation has been traditionally believed to be the predominant mechanism of kidney stone damage in fragmenting under high *E_p_* [9], [18], in which the fiber tip is placed in close contact with the stone surface [19], [20]. In contrast, the contribution of cavitation to kidney stone damage was largely disregarded due to the weak pressure generated by the collapse of the elongated vapor bubbles [19], [21]. However, we have recently demonstrated that cavitation plays a vital role in kidney stone dusting under low *E_p_* when the fiber tip is either not in direct contact with or scanning over the stone surface, resulting in a significant amount of the irradiated laser energy being absorbed by the interposing fluid [22], [23]. Despite this distinctly different and potentially paradigm-changing observation, the physical mechanism and associated optimal LL conditions for cavitation-driven kidney stone damage have not been elucidated.

Cavitation is a multiphysics process important in a broad range of engineering and biomedical applications [24], [25], [26]. Two primary mechanisms have been attributed to cavitation-induced damage on solid boundaries: 1) shock wave emission produced by the initial rapid expansion and ensuing violent collapse of the bubble [27], [28], and 2) high-speed liquid jet impact on the solid surface with resultant water hammer pressure or toroidal bubble formation and collapse [29], [30], [31], [32], [33], [34]. However, most previous studies were focused on inertial cavitation bubbles with insignificant vapor content generated by nanosecond (ns) pulsed lasers [31], underwater explosion [35], and lithotripter shock waves [24], [36], [37], [38]. In contrast, bubbles in LL are produced by the irradiation of long pulse (>70 µs) lasers under thermal confinement (yet without stress confinement), resulting in the formation of elongated pear-shaped bubbles of significant vapor content and weak acoustic emission (<20 bars) during bubble collapse [19], [21], [39]. At present, the relative contribution of photothermal ablation vs. cavitation damage to kidney stone dusting is still under debate [9], [20], [22], [23]; hindering technological advances and optimization of treatment strategies for KSD patients.

In this work, we seek physical insights into the mechanisms of material damage and removal produced by bubbles generated during the emerging clinical strategy of stone dusting in LL. First, we investigate laser energy partition in the fluid and solid domains experimentally and using model-based simulations. Second, we examine comprehensively the bubble dynamics and associated pressure transients generated near a solid boundary during LL using combined high-speed shadowgraph/photoelastic/total-internal-reflection imaging and hydrophone measurements. We further correlate detailed features of the jet impact vs. toroidal bubble collapse with the pressure transients produced and the resultant kidney stone damage. Third, we analyze numerically the fluid–structure interaction associated with the pressure impulse from the toroidal bubble collapse using a multiphysics model. Altogether, our results suggest that although less than one percent of the laser pulse energy is converted to the maximum bubble potential energy, the resultant shock wave-stone interaction driven by the intensified toroidal bubble collapse is the primary mechanism for cavitation erosion and stone damage during the dusting procedures using Ho: YAG lasers.

## 2 Materials and method.

### 2.1 Laser energy transmission measurement

All experiments were conducted using a Ho: YAG laser lithotripter (H Solvo 35, Dornier MedTech, Munich, Germany) under dusting setting (*E*_p_ = 0.2 J, *F* = 20 Hz) with a pulse duration of 70 μs (measured at the full width at half maximum (FWHM)). In some experiments, the laser delivery fiber (Dornier SingleFlex 400, NA = 0.26, 365 µm core diameter) was submerged in water and positioned perpendicularly to a 1 mm thick glass slide. A translation stage was used to control the fiber standoff distance (SD) from 0 to 1.5 mm from the surface of the glass slide. A light guide was positioned approximately 20 mm away on the opposite side of the glass slide in air to collect the transmitted light to an InGaAs photodetector (PDA10D, Thorlabs, Newton, NJ). Transmitted laser pulse energies were quantified by integrating the power profiles over time, and the mean relative pulse energies (n = 30) were normalized by the relative value measured at SD = 0 mm where no water absorption was assumed. The test was repeated using a higher *E*_p_ = 0.8 J in fragmenting mode [22] for comparison. No apparent damage or deformation to the slide was observed following the LL treatment.

### 2.2 *In vitro* laser lithotripsy treatment

BegoStone phantoms (50 × 50 mm lateral dimension, 5 mm thickness), a common kidney stone phantom with similar mechanical properties to human calculi, were prepared with a powder to water ratio of 5:2 as described in [22]. The BegoStone phantoms were pre-soaked in water for 24 h prior to the experiments. During the LL treatment, the laser fiber was placed perpendicular to the stone flat surface either in water or in air under SD = 0, 0.5, 0.75, and 1 mm. After the treatment, the resultant damage on the stone surface was examined by optical coherence tomography (OCT, OQ Labscope, Lumedica, Durham, NC) and quantified using in-house scripts written in Matlab (Mathworks, Natwickm, MA) [22], [23].

### 2.3 Dynamic shadowgraph/photoelastic imaging and acoustic emission measurement

**Fig. 1:**
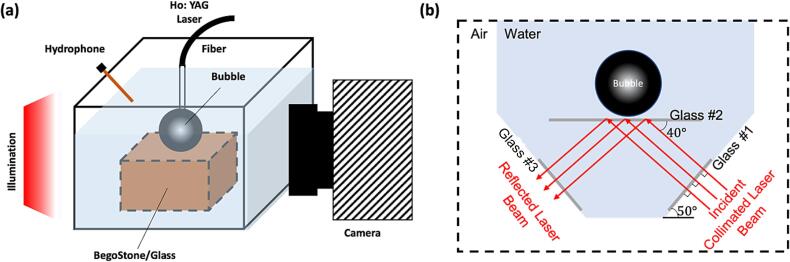
Schematic illustration of the experimental setup. (a) The high-speed shadowgraph imaging setup with hydrophone measurement. Note that the BegoStone/glass position will change based on the different viewing angles from the camera. (b) Schematic illustration of the total-internal-reflection imaging setup.

To study the highly dynamic process of the vapor bubble oscillation and bubble-solid interaction, we performed high-speed imaging to record the bubble shape dynamics accompanied by simultaneous hydrophone measurement (see Fig. 1a). The Ho:YAG laser induced vapor bubble was produced in an optical transparent water container (120×120×120 mm^3^, L×W×H) filled with distilled water. A 1 mm thick glass slide was aligned parallel to the beam propagation direction under side view and 45 deg view. The laser fiber was positioned perpendicular to the glass slide and the SD was selected as 0.25, 0.5, 0.75, 1.0, 1.5, and 3.0 mm. The transient vapor bubble dynamics and the bubble-solid interaction were recorded by using an ultra-high-speed camera (Kirana-M5, Specialised Imaging) operated at 0.2 to 5 million frames per second (fps) with backlighting provided by a 10-ns pulse laser illumination system (SI-LUX-640, Specialised Imaging). Furthermore, additional experiments were carried out by attaching a thin layer (0.5 mm) of hard BegoStone phantoms on a PSM-4 piece (90×8×20 mm^3^, L×W×H) to allow for surface damage test and simultaneous shadowgraph/photoelastic imaging under the same SD range used for the glass slide experiment. The pressure waveforms of the transient acoustic emissions were acquired by a needle hydrophone (HNC-1000, Onda) connected to a digital oscilloscope (HDO-6104, Teledyne LeCroy), which was placed 10 mm from the fiber tip to prevent damage to the sensor and avoid affecting the bubble dynamics. To synchronize the experimental system, a pulse delay generator (Berkeley Scientific, BNC model 555) was used to trigger the camera, the illumination system, and the hydrophone using the trigger output signal from the Ho: YAG laser lithotripter.

The recorded high-speed images were post-processed using ImageJ. The peak acoustic pressures were calculated based on the calibration data provided by the hydrophone manufacture and all the acoustic pressures were scaled to 1 mm separation distance from the fiber tip. For each pressure trace, when a pressure peak is larger than 30 % of the maximum pressure shown in the trace, it will be selected as a significant peak and used for further analysis.

### 2.4 Dynamic total-internal-reflection (TIR) imaging

A customized water container, including a trapezoidal part and a rectangular part, was used to establish the total-internal-reflection at the liquid/gas interface, as depicted in Fig. 1b. The illumination laser (SI-LUX-640, Specialised Imaging) beam was first expanded by a 40x microscope objective and then collimated by a convex lens (*f* = 75 mm). The collimated beam first transmitted through glass window #1 and projected on the target glass #2 with an incident angle of 50 deg, where the laser fiber tip was placed on the other side of the glass #2. Such an incident laser beam will be totally reflected when there exists a gas phase on the opposite side of glass #2, and transmitted through glass window #3 to the camera, triggered by the same setup used in the dynamic photoelastic/shadowgraph imaging. For the captured image, the bright “white” region corresponds to a glass/gas interface (i.e., the bubble region), and the dark region indicates the glass/water interface [40].

### 2.5 3D Monte-Carlo simulation

The Monte Carlo (MC) simulation was performed by a commercial software TracePro (TracePro XP and RayViz, LAMBDA) to study the dynamic laser energy distribution and absorption in both the fluid and solid for a Ho: YAG fiber laser. In the simulation, the fiber tip and BegoStone were immersed in water, and the SD between the fiber tip and the flat stone surface was 0.5 mm. The simulation was performed in three-dimension with a total volume of 2.5×2.5×3 mm^3^ and a voxel size of 0.01×0.01×0.04 mm^3^. The optical properties of water and BegoStone at 2080 nm are listed in the supplemental material (Table S1). The laser beam was directed perpendicularly to the stone surface, with a total pulse duration of 150 μs. The power profile was grouped into 30 even segments and the total optical fluence and absorbance during each segment were simulated and recorded individually. The dynamic geometry of the vapor bubble extracted from the high-speed images for SD = 0.5 mm at *E_p_* = 0.2 J was adapted in the MC simulation with the inner volume considered as vacuum and the optical absorption by the vapor inside the bubble ignored.

### 2.6 Thermal ablation simulation

The thermal ablation of the laser was simulated using the heat conduction model described in [41], which reads briefly as,

(1) $$\rho C_{p}\frac{\partial T}{\partial t}-k\nabla T=f,$$

where ρ, $C_{p}$, k and f are the mass density, specific heat capacity, thermal conductivity and source term related to the laser irradiation. The transient heat conduction was simulated using an embedded finite-element method, which is detailed in [41]. In the simulation, the material was only ablated when the incoming laser energy exceeds the thermal resistance of the material, and the solid temperature reaches the melting temperature. A 2D axisymmetric configuration ($\Omega =\left[0,5\right]\times \left[0,5\right]$ mm, where Ω is the domain of the solid) was adopted with laser pulses assumed to be interacting with the solid domain from the top and homogeneous Neumann boundary conditions were applied to the rest boundaries [41]. The model was calibrated against the experimental data obtained from wet BegoStones treated in air by modulating the percentage of laser energy absorbed by the stone material.

### 2.7 Coupled laser-fluid simulation

We employed a coupled fluid-laser framework with phase transition and interface tracking to model the bubble initiation and expansion induced by a Ho: YAG fiber laser [42]. Assuming that the fluid is compressible and inviscid, the two-phase fluid flow is governed by the Euler equations, which is given as

(2) $$\frac{\partial W}{\partial t}+\nabla ∙F=\nabla ∙G,$$

with

$$W=\left[\begin{array}{c} \rho \\ \rho V\\ \rho E \end{array}\right],\;F=\left[\begin{array}{c} \rho V\\ \rho V⊗V+pI\\ \left(\rho E+p\right)V \end{array}\right],G=\left[\begin{array}{c} 0\\ 0\\ -q_{r} \end{array}\right]$$

where V is the fluid velocity, E is the total energy per unit mass ($E=e+\frac{1}{2}{\left|V\right|}^{2},e$ is the internal energy per unit mass), $q_{r}$ is the radiative heat flux.

The laser radiation equation is derived based on the energy conservation and the assumption,

(3) $$\nabla ∙\left(Ls\right)=\;-\mu_{\alpha }L,\;and\;q_{r}=Ls$$

where L, $sand\;\mu_{\alpha }$ are the laser radiance, direction, and absorption coefficient, respectively. Both the Euler equations and the laser irradiation equation were discretized based on the finite volume method. The exact two-phase Riemann solver was applied to solve the governing equations, referred as FIVER [43], [44], [45]. The gas–liquid interface was tracked by the level set method and an embedded boundary method was utilized to impose the laser boundary conditions. The phase transition process was accomplished when the latent heat reaches with the vaporization temperature ranging from 373.15 K to 533.15 K under the assumption of homogeneous cavitation nucleation [46]. For the computational setup, a cylindrical domain (6 × 12 mm, radius × length) was specified with the bulk fluid set as liquid water under ambient condition (1 atm, 20 °C). The laser wavelength, fiber diameter, and the laser power profile measured in air from the experiment were used to construct the temporal power profile of the laser source. The laser absorption coefficient are 2.42 mm^−1^ and 0.001 mm^−1^ for the liquid water and vapor [9], respectively. The simulation was carried out on a two-dimensional Cartesian mesh by assuming the axisymmetric condition with a characteristic element size of 25 µm.

### 2.8 COMSOL modeling

A simplified model mimicking the shock wave-solid interaction was adopted, in which the source of shock wave emitted from the bubble collapse spot was modeled by a monopole point source as described in [47]. The numerical model was constructed in COMSOL Multiphysics 5.6 (Burlington, USA) using the Acoustic-Structure Interaction, Transient Multiphysics Interface in the acoustic module. The mechanical properties of quartz and hard BegoStone were listed in the supplemental material (Table S2). An axisymmetric model was considered with the *z*-axis of the cylindrical coordinate system aligned normal to the solid surface and pointed to the monopole source. Both the fluid and solid domains were discretized by the second order triangular Lagrangian elements. The detailed description of the model was shown in [47].

## 3 Results

### 3.1 Energy partition and distinct features of cavitation damage in Ho:YAG LL

**Fig. 2:**
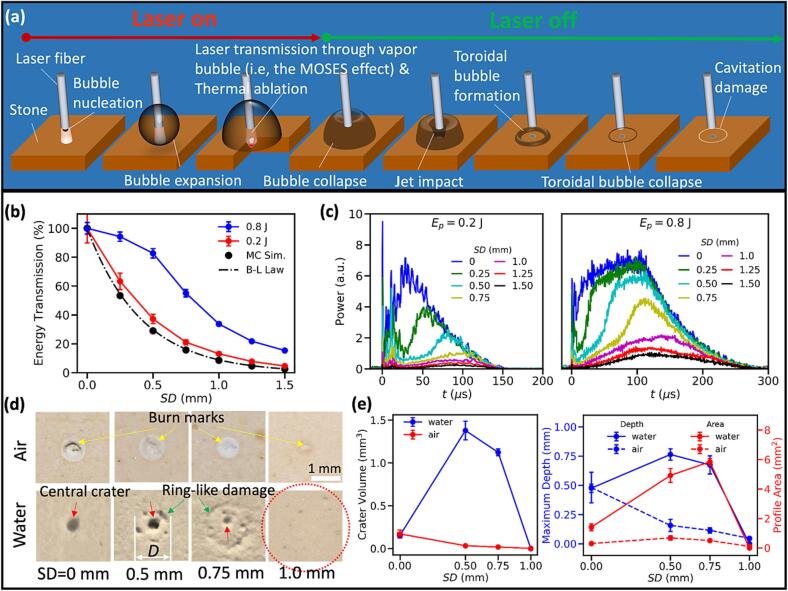
Important physical processes in laser lithotripsy (LL), laser energy transmission, and distinct features of stone damage produced by dusting treatment. (a) Schematic diagram of the various physical processes involved in LL. (b) Ho:YAG laser energy transmission in water measured at *E_p_* = 0.2 J and 0.8 J under various SDs, together with the energy transmission predicted by Beer-Lambert (B-L) law and Monte Carlo (MC) simulations without phase change (i.e., bubble formation). (c) Transmitted laser pulse power profile under different SDs at *E_p_* = 0.2 J and 0.8 J, respectively. (d) Representative damage patterns produced on wet BegoStone surfaces after 20 pulses either in air or water at *E_p_* = 0.2 J, SD = 0, 0.5, 0.75, and 1 mm. *D*: the outer diameter of the ring-like damage. Yellow arrow points to the burn marks around the thermally ablated central crater in air, red arrow points to the central crater at the laser beam projected area, and green arrow marks the ring-like damage surrounding the central crater produced in water. The dashed red circle indicates the maximum equivalent bubble in the experiment. (e) Crater volume (left) and maximum depth and surface profile area (right) vs. SD produced by dusting treatment in air or in water after 250 pulses at *E_p_* = 0.2 J and PRF = 20 Hz. (For interpretation of the references to colour in this figure legend, the reader is referred to the web version of this article.)

The standoff distance (SD) between the fiber tip and the stone surface critically determines the laser energy partition in the interposing fluid and target stone, and thus will profoundly impact the physical mechanism responsible for material damage in LL [20], [22], [48]. Fig. 2a illustrates schematically and sequentially three important stages of LL: 1) laser absorption in water and initiation of vapor bubble formation at the fiber tip, 2) bubble expansion and laser transmission through the interior of the vapor bubble (i.e.*,* the Moses effect, [49]) to reach the target stone for photothermal ablation, and 3) primary bubble collapse following the cessation of laser irradiation with resultant liquid jet impact and toroidal bubble formation/collapse that may lead to cavitation erosion.

We measured the laser energy transmission in water at different SDs and *E_p_* levels. Due to the Moses effect, greater energy transmission was detected compared to the predictions by the Beer-Lambert law [50] and Monte Carlo simulations for laser absorption in water without bubble formation (Fig. 2b). For example, at SD = 0.5 mm (Fig. 2c), the energy transmission is much more efficient at high *E_p_* (0.8 J) for fragmenting (>80 %) than at low *E_p_* (0.2 J) for dusting (<40 %). These significant differences may contribute to the shift of the predominant mechanism of stone damage from the conventional theory of photothermal ablation during fragmenting to recently uncovered cavitation-driven damage in stone dusting [22], [23].

More importantly, distinctly different damage patterns were produced on the BegoStone (i.e., a phantom for kidney stones [50]) surfaces during dusting at various SDs after 20 pulses (Fig. 2d). In air, when the laser energy was absorbed directly by the stone surface, a circular crater right underneath the fiber tip surrounded by discolored burn marks were produced by photothermal ablation. The crater size and volume were found to decrease with SD from 0 mm to 1.0 mm, in association with the concomitantly reduced laser fluence delivered to the stone surface [23]. In comparison, for stones treated in water, a ring-like damage pattern emerged in the SD range of 0.5–0.75 mm around the central crater with no discolored burn marks in between, leading to significantly augmented total material removal. Moreover, while the central crater size decreased with SD, the diameter (*D*) of the ring-like damage increased from SD = 0.5 mm to SD = 0.75 mm. It is worth noting that no ring-like damage was produced at either SD = 0 or 1 mm, which may correlate with the dissimilar laser-fluid-stone interaction and resultant bubble dynamics and collapsing patterns produced at various SDs [23].

Even though more laser energy was delivered to the stone in air, the maximum crater volume after 250 pulses were produced in water at SD = 0.5 mm, which is about 7-fold its counterpart in air at SD = 0 mm (Fig. 2e). The damage crater produced in water has greater depth and surface profile area, and thus substantially increased crater volume. These unique damage features are in direct contradiction to the conventional theory of photothermal ablation in LL [19], raising questions about the energy partition and associated physical mechanisms truly responsible for stone dusting in LL.

**Fig. 3:**
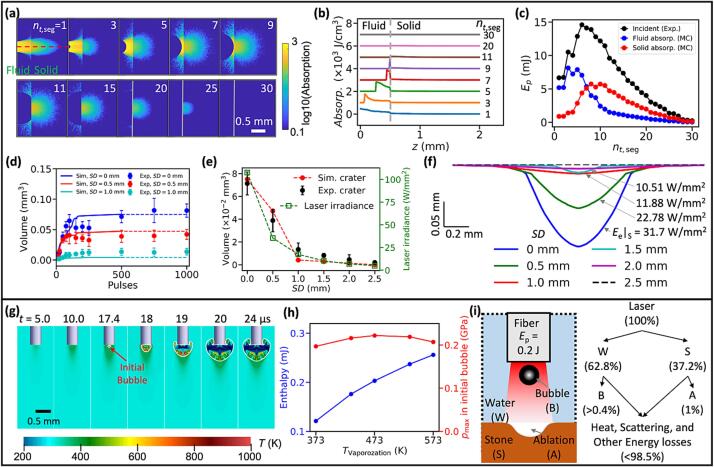
The laser absorption by the fluid and solid in LL under dusting, *E_p_* = 0.2 J. (a) MC simulation of deposited laser pulse energy in water and stone under SD = 0.5 mm. The laser energy emitted from the fiber tip is equivalently divided into 30 segments with a time duration of 5 μs, and the segment number *n_t_*_,seg_ = *t*/*t*_sep_ (where *t*_sep_ = 5 μs is the time segment length used in the Monte Carlo simulation) = 1, 2, …, 30. (b) The distribution of laser absorption along the central line (red dash line in the first frame) in (a), plotted in the linear scale. (c) The time history of incident laser energy, and energy absorbed by the interposing fluid and the solid. Note that the absorbed energy by the solid and fluid are integrated over the entire volume. (d) Crater volume for varying SDs. Simulation results are obtained for 1 % the incident laser energy. (e) Experimental and simulated crater volumes as well as the laser irradiance on the intact stone surface under varying SDs at *E_p_* = 0.2 J. (f) Simulated crater profiles after 500 pulses under varying SDs. *E*_e_|_S_ is the laser irradiance at the saturated crater surface. (g) Temperature evolution and bubble initiation at the early stage of laser irradiation. (h) The enthalpy of vaporization required for bubble initiation and the maximum pressure inside the initial bubble for varying vaporization temperatures of water. (i) A comparison of the energy partition during laser irradiation for SD = 0.5 mm at *E_p_* = 0.2 J. (For interpretation of the references to colour in this figure legend, the reader is referred to the web version of this article.)

Bubble dynamics in LL can significantly influence the energy partition in the fluid and solid. As shown in Fig. 3a–c, during the expansion and before the advancing bubble apex reaches the stone surface (at the segment number *n_t,seg_* = *t*/*t*_sep_ = 5 where *t*_sep_ = 5 μs is the time segment length used in the Monte Carlo simulation), most of the laser energy is absorbed by the intervening water with negligible energy delivered to the stone. Thereafter, the remaining laser pulse energy, transmitting through the vapor tunnel, is mostly absorbed and scattered by the stone material with a portion dissipated in the nearby fluid. It is worth noting that the laser scattering can reach a broader region both in the fluid and solid beyond the beam irradiating path (Fig. 3a).

It has been shown that during LL when the collapse of the vapor bubble toward the stone surface was mitigated, the resultant craters produced in water resembled closely to those in air [23]. We thus numerically simulated the thermal ablation process of the BegoStone material in air during dusting treatment [51] and found that only 1 % of the incident *E_p_* was required to produce the stone damage observed experimentally (Fig. 3d). Moreover, the model simulations captured the general trend in the photothermal ablation process, which is characterized by an initially rapid increase in crater volume and depth followed by a progressive saturation after 200 to 250 pulses [23]. Combined with the MC simulation results shown in Fig. 3a, these findings suggest that majority of the laser *E_p_* delivered to the stone material was either scattered or reflected while only a small portion was absorbed at the bubble-stone interface to increase the temperature at the irradiated boundary beyond the melting point of the stone material. Physically and in the simulations, the saturation in the photothermal ablation process may result from multiple factors, including: 1) the laser irradiance at the progressively ablated stone surface will decrease as the effective fiber tip to the remaining stone surface distance becomes larger with concomitantly enlarged surface area under laser irradiation [51], and 2) the discolored burn marks deposited on the stone surface (see Fig. 2d) may significantly change the optical absorption coefficient of the stone material [52]. The first speculation is supported by an apparent threshold in laser irradiance (∼5.79 W/mm^2^ at SD = 2.5 mm), beyond which the thermal ablation is negligible. This experimental observation is reasonably matched with the model-calculated laser irradiance values at the saturated crater surfaces produced under different SDs (Fig. 3e and f).

Next, we modeled the laser-fluid interaction and resultant vapor bubble formation and expansion in bulk fluid (Fig. 3g). Based on the theory of superheating that accounts for different vaporization temperatures of water ranging from 373.15 to 673.15 K [53], only 0.12–0.26 mJ of latent heat (or enthalpy) of vaporization is required for the phase change, corresponding to 0.06–0.13 % of the *E_p_*. However, to reach the vaporization temperature (e.g., 373.15 K at 17.4 µs), our model simulation suggests that 38.95 mJ of the laser energy (or 19.5 % of *E_p_*) will be absorbed in the water surrounding the fiber tip to initiate the bubble formation (Fig. 3g). Following the phase transition, the pressure inside the initial bubble might reach up to 0.15～0.22 GPa (Fig. 3h), forging a primary driving force for the subsequent rapid expansion of the bubble. Thereafter, the laser irradiation will continue to subcritically heat the liquid near the bubble apex without phase change during the ensuring expansion until the laser reaches the stone surface.

A quantitative summary of the energy partition in the fluid and solid is shown in Fig. 3i. At a typical SD = 0.5 mm for dusting treatment (see Fig. 2b), 62.8 % of the total incident laser energy is absorbed by the intervening water, while 37.2 % is transmitted to the target stone. In the fluid, a minimal amount of energy (∼19.5 %) is needed to initiate the bubble, what will be subsequently converted into energies associated with possible acoustic emission, viscous dissipation and heat generation, kinetic energy in the fluid, and potential energy of the vapor bubble at maximum expansion (∼0.4–0.7 %). In the stone, while only 1 % of the laser energy may be used to produce thermal ablation in water, the rest of the transmitted laser energy is scattered in the stone material, eventually absorbed to rise temperature sublethally in the bulk material, and/or re-absorbed by the fluid near the irradiated stone surface (see Fig. 3a).

### 3.2 Distinct features of the bubble dynamics generated by Ho:YAG LL in dusting

**Fig. 4:**
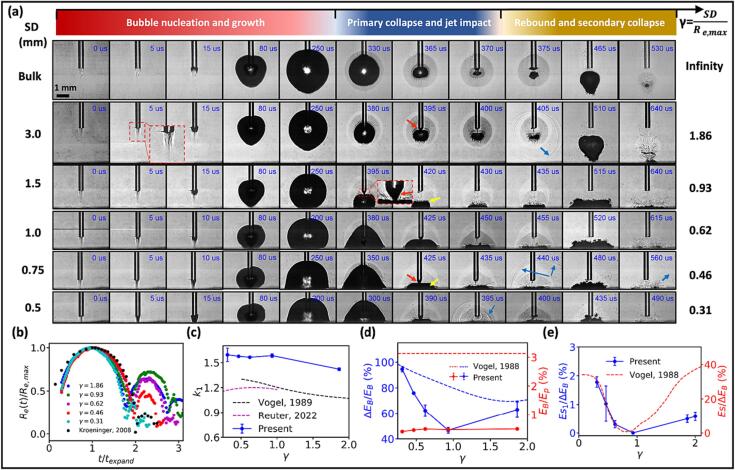
Cavitation bubble dynamics induced by Ho:YAG laser lithotripsy at 0.2 J pulse energy near a solid (glass) boundary. (a) Side view images of the bubble dynamics for SD = 0.5, 0.75, 1.0, 1.5, and 3.0 mm, as well as in bulk fluid. The arrows indicate the cone bubble (red), the toroidal bubble (yellow), and the shock waves (blue), respectively. (b) Equivalent bubble radius, R_e_(t) vs. time t under different γ, which is the dimensionless standoff distance. R_e_(t) is normalized by the maximum equivalent bubble radius (R_e,max_, the value of which under different SDs(γ) is shown in Supplementary Information) and t is normalized by the Rayleigh collapse time (T_c_, $T_{c}=0.915R_{e,max}\sqrt{\frac{\rho }{\left(p_{\mathrm{stat}}-p_{v}\right)}}$, where $R_{e,max}$ is the maximum equivalent bubble radius, ρ is the density of the liquid, p_stat_ and p_v_ are the static pressure and vapor pressure of the liquid, respectively) of a spherical bubble. The black square shows the time history of bubble radius calculated from the collapse of a spherical bubble induced by ns pulsed laser in water [59]. (c) The prolongation factor k_1_ for the bubble collapse vs. γ. (d) Percentage of the bubble energy loss (ΔE_B_/E_B_) and laser pulse energy conversion into the bubble potential energy (E_B_/E_P_) during the primary collapse. Here ΔE_B_ = E_B_-E_B,1_, E_B_: maximum bubble potential energy following the initial expansion, $E_{B}=\frac{4}{\pi }\left(p_{\mathrm{stat}}-p_{v}\right)R_{e,max}^{3}$, E_B,1_: maximum potential energy of the bubble after the first rebound. (e) Percentage of acoustic emission energy ($E_{s1}$) normalized by ΔE_B_ during the primary collapse vs. γ. The red dash curve is the percentage of acoustic energy $\left(E_{s}\right)$ normalized by ΔE_B_ during the primary collapse of ns-laser generated bubble, replicated from [55]. (For interpretation of the references to colour in this figure legend, the reader is referred to the web version of this article.)

The overall bubble dynamics in LL can be categorized into three distinct phases: 1) bubble nucleation and growth, 2) primary bubble collapse with jet impact, toroidal bubble formation and collapse, and 3) bubble rebound and secondary collapse (Fig. 4a). At the onset of laser irradiation (0–5 μs), a plume of superheated fluid is observed from the visible hot filaments underneath the fiber tip, which leads to the initiation of an aggregated vapor bubble through phase transition [16]. During the bubble growth (up to 250 μs), the remaining laser pulse irradiation will continue to superheat the fluid near the apex of the pear-shaped vapor bubble, driving a prolonged and elongated expansion [13]. At a large SD of 3.0 mm, the maximum bubble expansion is reached without contacting the solid boundary. The bubble then collapses axially and radially, forming a jet (395 μs) and a toroidal-shaped minimum volume (405 μs). Subsequently, the bubble rebounds (580 μs) and collapses again while translating toward the solid boundary (640 μs). As SD decreases (≤ 1.5 mm), the vapor bubble reaches the solid boundary during the expansion, which facilitates the transmission of the remaining laser pulse energy to the solid. A small cone bubble [54] is formed at the fiber tip during the primary collapse of the bubble in the SD range of 0.5–3 mm with resultant weak shock wave emissions, in comparison to ns-laser induced bubble collapse [29].

Several distinct features of bubble dynamics in LL become clear when the results are plotted against non-dimensional standoff distance *γ* $=SD/R_{e,\;\max}$ where $R_{e,\;\max}$ is the maximum equivalent radius of the bubble produced in bulk fluid. First, due to the long pulse duration, LL-induced vapor bubbles experience a weaker primary collapse with a larger minimal volume, followed by a stronger rebound (Fig. 4b), in comparison with the collapse of a spherical bubble produced by ns-lasers [55]. The excessive vapor [21] or non-condensable gas contents inside the bubble [56], [57] may dampen the bubble collapse in LL. Second, the non-spherical geometry of LL-generated vapor bubbles have a more pronounced influence on bubble behavior near a solid boundary, compared to their spherical counterparts [55], [58]. This is manifested by the significantly increased prolongation factor $k_{1}=t_{1,LL}/{2T}_{c}$ where *t_1, LL_* corresponds to the time duration from the inception to the primary collapse of LL-induced bubble, and *T_c_* is the Rayleigh collapse time of a spherical bubble of equivalent maximum volume (Fig. 4c). Third, the energy conversion efficiency in bubble generation and growth is low, and so is the energy dissipation rate during the primary collapse of LL-induced bubbles (Fig. 4d). At large $\gamma \left(=1.86\right),$ less energy loss is associated with acoustic emission during the dampened bubble collapse (Fig. 4e) compared to the scenario at γ<0.3. Interestingly, however, the minimal acoustic emission was still observed around γ=0.9 during LL, as it is in the case for ns-laser generated bubbles when the bubble potential energy (*E_B_*) was maximally converted into the kinetic energy of the liquid jet [29], [55]. Thereafter, the intensity of the bubble collapse increased rapidly with reduced γ(<0.9), with a remarkable agreement in the trend of the progressively intensified pressure transients for either LL- or ns laser-induced bubble collapses closer to the solid boundary (Fig. 4e). It should be noted that the ratio of acoustic energy over the potential energy loss during the primary collapse (*E_s1_/ ΔE_B_*) for LL-induced bubble is about 1/20 of its counterpart for ns laser-induced bubble, indicating again that the collapse of LL-induced bubbles is significant dampened.

**Fig. 5:**
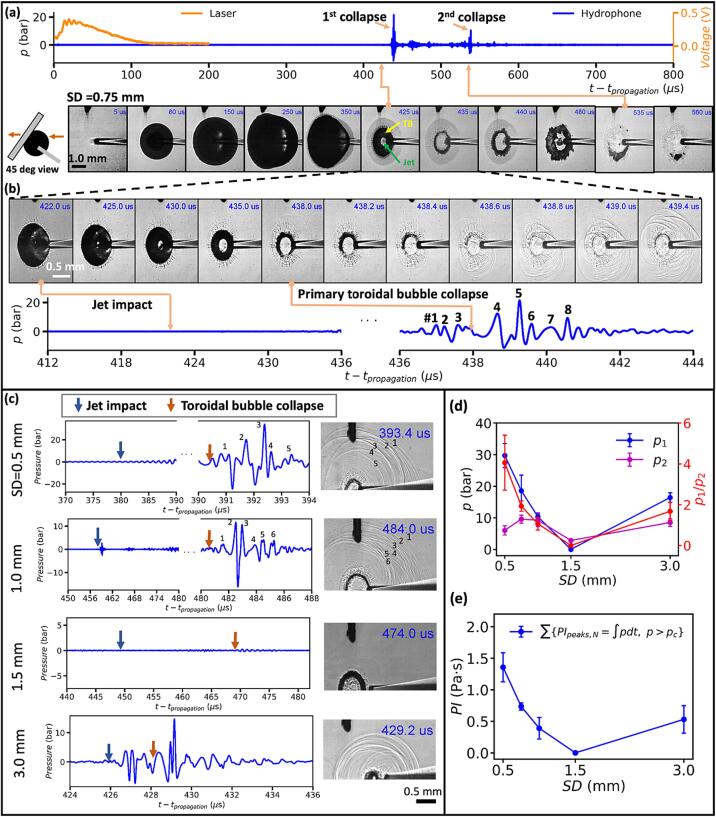
High-speed shadowgraph images of cavitation bubble dynamics generated by Ho:YAG laser at 0.2 J and 20 Hz together with simultaneous hydrophone recordings. (a) Shadowgraph images captured at 200,000 fps and acoustic pressure transients associated with the full bubble dynamics produced at SD = 0.75 mm. The diagram next to the first image frame shows the camera angle with the arrows indicating the beam path for back-lit imaging. (b) Detailed high-speed shadowgraph images captured at 5 million fps and associated acoustic transients produced by the primary bubble collapse at SD = 0.75 mm showing jet impact, toroidal bubble formation and collapse with shock wave emissions. (c) Simultaneous acoustic transients measured by hydrophone (left) and snapshot of the toroidal bubble collapse (right) produced under various SDs. *t*_propagation_: the traveling time (7.65 ± 0.65 μs) of the acoustic wave from the collapse spot to the hydrophone. The pressure peaks in the acoustic transients and corresponding shock waves in the shadowgraphs are labeled. (d) Variations of the maximum peak pressures from the primary bubble collapse (*p*_1_) and secondary bubble collapse (*p*_2_), as well as the ratio of *p*_1_/*p*_2_ vs. SD. (e) The variation of pressure impulse (PI) with SD. All the pressures are corrected to values estimated at 1 mm distance from the collapse center using the 1/*r* relationship for a spherically diverging shock wave.

The details in the bubble collapse have been resolved using high-speed shadowgraph imaging at 5 Mfps (million frame per second) from the 45° angle together with simultaneous hydrophone measurements. For example, at SD = 0.75 mm (Fig. 5a), no significant pressure transients are detected either during the laser irradiation (0–150 μs) or upon the jet impact (425 μs, represented by the central bright region surrounded by the dark toroidal ring). In contrast, immediately following the jet impact, a toroidal bubble is formed (Fig. 5b and Supplementary Movie S1), which collapses in about 13 μs, starting from one lateral spot (438.0 μs) off the fiber axis and moving along the torus to the distal end of the remaining bubble (438.8 μs) through a progressively intensified collapsing process, creating concomitantly a burst of shock wave emissions. Specifically, the weak shock waves (peaks #1∼#3) produced by the initial collapse will intensify the subsequent collapse of the remaining torus bubble (438.2–438.8 μs), generating stronger shock wave emissions (peaks #4∼#5). Thereafter, the torus bubble will rebound and undergo the secondary collapse (Fig. 5a, 535 to 560 μs).

A careful examination of the primary bubble collapse at various SDs confirms that the shock wave emissions are indeed generated by the toroidal bubble collapse, but not by the jet impact (Fig. 5c). The peak pressure (*p*_1_) associated with the primary toroidal bubble collapse decreases rapidly with SD, from 30 bars at SD = 0.5 mm (γ= 0.31) to a minimal value of 0.25 bars at SD = 1.5 mm (γ= 0.93), before increasing again to 16.4 bars at SD = 3.0 mm (γ= 1.86). In comparison, the peak pressure (*p_2_*) associated with the secondary toroidal bubble collapse is overall weaker and less variable with SD (Fig. 5d). A similar trend is observed in the variation of the pressure impulse (*PI*) with SD (Fig. 5e). Further, the largest ratio of *p*_1_/*p*_2_ is observed at SD = 0.5 mm (γ= 0.31), corresponding to the highest percent of energy conversion into shock wave emission and pressure impulse. This finding is consistent with the significantly increased stone damage observed experimentally at such fiber tip-stone distance [23].

**Fig. 6:**
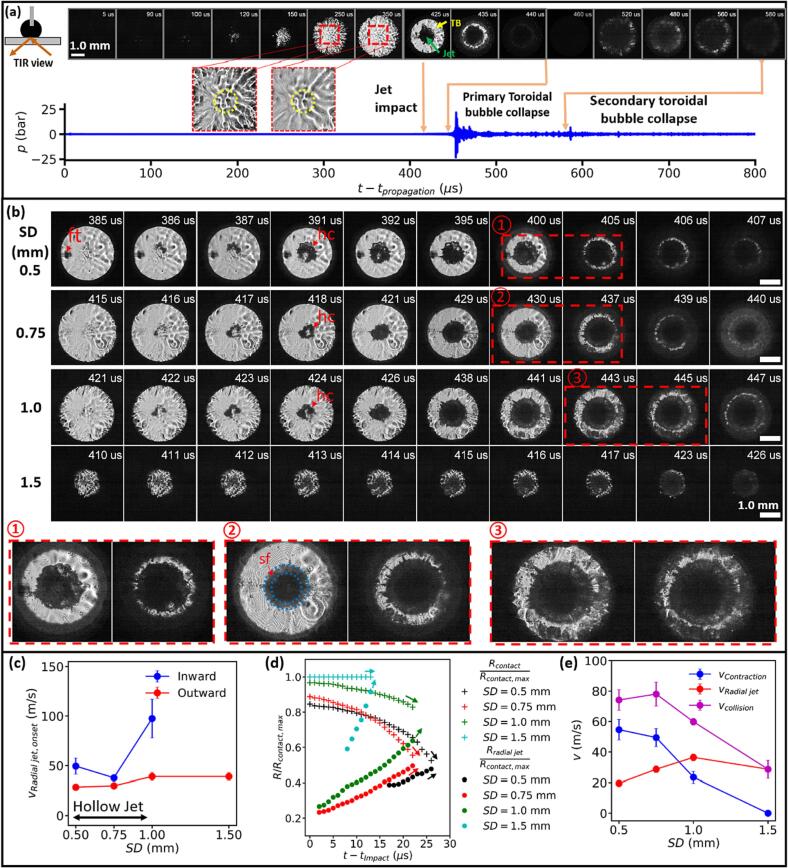
Features of the bubble collapse with jet impact, toroidal bubble formation and collapse against a glass boundary captured by total-internal-reflection (TIR) imaging during stone dusting (0.2 J and 20 Hz) in Ho:YAG laser lithotripsy. (a) TIR images captured at 200,000 fps and associated acoustic transients captured during the full lifespan of the cavitation bubble, produced at SD = 0.75 mm. The diagram before the first frame shows the camera angle with the arrows indicating the light beam path for the TIR imaging. “TB” denotes the toroidal bubble. The yellow dotted circles in the two inset images indicate the laser irradiation zone on the glass surface. (b) TIR image sequences captured at 1 million fps for SD = 0.5, 0.75, 1 and 1.5 mm. “ft” denotes the fiber tip, “hc” denotes the hollow core of the liquid jet upon initial impact, and “sf” denotes the splashing flow driven by the outward radial flow formed in the late stage of the jet impact. (c) The average lateral speed of the inward and outward radial flows at the onset of the liquid jet impact. (d) The variations of the maximum radius of the bubble (i.e., outer wall of the torus bubble) in contact with the solid boundary and the maximum radius of the radial jetting flow (i.e., inner wall of the torus bubble) with SD. The arrows indicate the trend of the radius variations with SD. (e) The variations of the radial lateral speeds of the bubble wall contraction (*v*_contraction_) outward radial jetting glow (*v*_radial jet_), and the collision speed of the toroidal bubble (*v*_collision_ = *v*_contraction_ + *v*_radial jet_) on the solid boundary. (For interpretation of the references to colour in this figure legend, the reader is referred to the web version of this article.)

Using total internal reflection (TIR) imaging [40], [60], we further analyze the details of jet impact and progressive collapse of the torus bubble on the solid boundary (Fig. 6). In the TIR images, the solid–gas and solid–liquid interfaces can be distinguished by the bright and dark regions, respectively. As such, the bubble expansion upon contacting the solid boundary is observed, for example, at SD = 0.75 mm, starting at 90 μs and continues to the maximum expansion at–350 μs (Fig. 6a). Subsequently, the bubble collapses with the outer rim of the bubble-solid contact area shrinking inward while the dark circle at the center produced by the jet impact from 425 μs expanding concomitantly outward, forming the toroidal bubble, as shown in Fig. 5. The opposite movement of the inner and outer walls of the torus bubble drives its collapse with instability demonstrated by the irregular wall geometry (425–435 μs). Again, hydrophone measurements confirm that the liquid jet impact produces negligible pressure transient, while the two bursts of shock wave emission correlate with the primary and secondary collapses of the toroidal bubble.

Several distinct features in the bubble-solid boundary interaction are noted below. First, after the maximum bubble expansion under small SD = 0.5 to 1.0 mm, there is still a thin liquid layer separating the bubble interior from the solid boundary, especially around the rim of the bubble-solid contact area (Fig. 6b and Supplementary Movie S2–S5). This observation is inferred from the interference fringes in the bright circles [40]. Second, the initial contact of the liquid jet with solid boundary is patchy and irregular with a hollow core, presumably formed in association with the cone bubble produced at the fiber tip (see Fig. 4a). With the jet contact area(s) initially moving both inward and outward radially within 4–5 μs upon touch down, the hollow core of the liquid jet will effectively reduce the outward expansion of the lateral flow that drives the toroidal bubble formation and collapse. As shown in Fig. 6c, the inward speed of the jetting flow is uneven and varies significantly in the range of 38 m/s (SD = 0.75 mm) to 98 m/s (SD = 1.0 mm). In contrast, the outward speed of the jetting flow increases steadily and slowly with SD in the range of 29 m/s to 39 m/s. Third, splashing flows (denoted as “sf”), indicated by the grey ring ahead of the central dark region, are observed during the latter stage of the jet impact, similar to ns laser-induced bubble collapse near a solid boundary [29], [32].

It is worth noting that instabilities during the toroidal bubble collapse are frequently observed. Due to imprecise alignment of the fiber tip with the solid boundary, axisymmetry is not achieved during the liquid jet impact, as indicated by the non-concentric geometry of the contact rings. As a result, the toroidal bubble collapses asynchronously, leading to the development of finger-like instabilities and disintegration of the torus bubble into multiple segments (see zoom-in images in Fig. 6b). As the SD increases from 0.5 mm to 1.5 mm, the outward expansion of the radial jetting flow in the central region will increase rapidly while the inward contraction of bubble wall is gradually slowed down to a transitory pause at SD = 1.5 mm (Fig. 6d). Consequently, at small SDs (0.5–0.75 mm) high collision speed of the toroidal bubble (*v*_collision_) is produced, driven by the combination of a strong bubble wall contraction with an increasing radial jet expansion. In comparison, at large SDs (1.0–1.5 mm) *v*_collision_ will decrease primarily due to a gradually stagnated wall contraction with limited radial jet expansion.

**Fig. 7:**
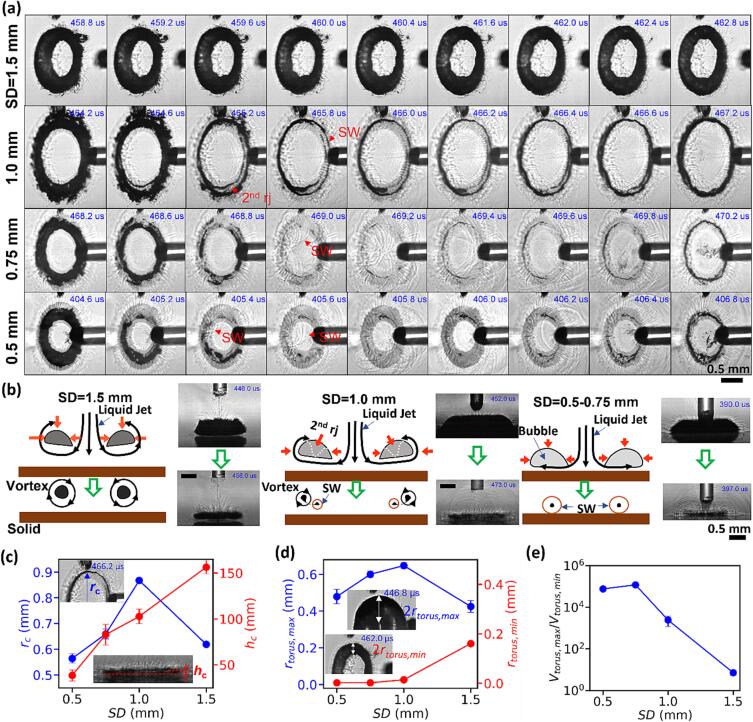
The final stage of the primary toroidal bubble collapse at different standoff distances (SDs). (a) Shadowgraph images captured at 5 Mfps from 45° view angle for SD = 0.5–1.5 mm. ‘SW’ denotes the shock waves, “2nd rj” denotes the secondary ring jet. (b) Schematic diagrams and shadowgraph images captured from the side view showing three different patterns of the toroidal bubble collapse (upper portion: initial stage of formation and collapse; lower portion: final collapsing stage), including 1) stagnated contraction with strong vortex formation (SD = 1.5 mm), 2) unstable contraction with weak vortex, secondary ring jet and double ring formation (SD = 1.0 mm), and 3) rapid volumetric contraction without vortex formation, leading to multi-segment breakup and collapses (SD = 0.5–0.75 mm). (c) The variations of the minimal radii (*r_c_*) and average height ($h_{c}$) of the torus bubble during its final stage of collapse with SD. (d) The variations of the maximum and minimum radii for the cross-sectional area of the torus bubble with SD. (e) The variation of the volumetric compression ratio (*V*_torus, max_ / *V*_torus, min_) vs. SD during the primary toroidal bubble collapse where $V_{torus}=2\pi^{2}r_{c}r_{torus}^{2}$.

Next, with these insights, we utilize the maximum spatiotemporal resolutions (5Mfps and 3.5 μm pixel resolution) of our experimental system to analyze the primary collapse of the toroidal bubble from both the 45°- and side-view angles (Fig. 7). It should be noted that the general bubble dynamics produced at different SDs are already shown in Fig. 4a. Here, we highlight several distinct features that can only be resolved at high frame rates to facilitate the physical interpretation of the results. First, at SD = 1.5 mm, most of the laser pulse energy is absorbed in the fluid, leading to high vapor/gas content generated and accumulated inside the bubble while only a small contact area is established with the solid boundary near the final stage of the bubble expansion (see Fig. 6a). As a result, the collapse of the toroidal bubble is substantially cushioned by its high vapor content with stagnated contraction near its minimal volume (e.g., see 462 μs in Fig. 7a at SD = 1.5 mm). From the side view, it can be seen clearly that a thin liquid layer separates the toroidal bubble from the solid boundary, levitating the torus bubble by strong circulating vortices (Fig. 7b, see also from 45^0^ view angle in the Supplementary Movie S6), which may prevent its continued shrinkage due to centrifugal effect [61], [62]. In comparison, at SD = 1.0 mm, the bubble contact area with the solid boundary is significantly enlarged with the resultant bubble torus translating closer to the solid surface (Fig. 7a and c, see also Fig. 4a). As a result, more laser energy is partitioned to the solid and less in the fluid, producing less vapor content inside the bubble and also a weaker vortex flow. Subsequently, the collapse of the toroidal bubble is less cushioned and more rapid with fast contractions, leading to instabilities developed early on and presumably the formation of a secondary ring jet (see illustrative diagram in Fig. 7b for SD = 1.0 mm), which created a double ring structure during the final stage of the collapse (see 464.6–465.8 μs in Fig. 7a at SD = 1.0 mm, also shown in Supplementary Movie S7). Furthermore, as SD decreases to 0.75 mm and 0.5 mm, the toroidal bubble collapses even closer to the solid boundary without vortex formation (Fig. 7b and c, see also Supplementary Movie S8 and S9). The final collapse is more violent due to the pure volumetric compression of the gaseous content of the bubble torus, leading to its disintegration into multiple segments, and subsequently the emission of multiple shock waves along the circumference (Fig. 7a).

Quantitatively, the radii ($r_{c}$) of the toroidal bubble near the final stage of toroidal collapse are 0.56 and 0.66 mm for SD = 0.5 and 0.75 mm, which match well with the size of the ring damage (Fig. 2e). The average height ($h_{c}$) of the toroidal bubble during its final stage of the collapse decreases from 165 µm at SD = 1.5 mm to 35 µm at SD = 0.5 mm (Fig. 7c), indicating that a higher acoustic energy flux can be exerted on the solid boundary by the toroidal bubble collapse at small SDs. During this period, the maximum radius (*r*_torus, max_) for the cross-sectional area of the torus bubble reaches a maximum at SD = 1 mm, while its minimal value (*r*_torus, min_) decreases exponentially from 160 μm at SD = 1.5 mm to–3.5 μm at SD = 0.5 and 0.75 mm (Fig. 7d). Consequently, the volumetric compression ratio (*V*_torus, max_/*V*_torus, min_) of the toroidal bubble collapse is 7.5 × 10^4^ at SD = 0.5 mm and 1.2 × 10^5^ at SD = 0.75 mm, which is four orders of magnitude greater than their counterparts at SD = 1.5 mm (Fig. 7e).

### 3.3 Relationship between the vapor bubble dynamics and stone damage

**Fig. 8:**
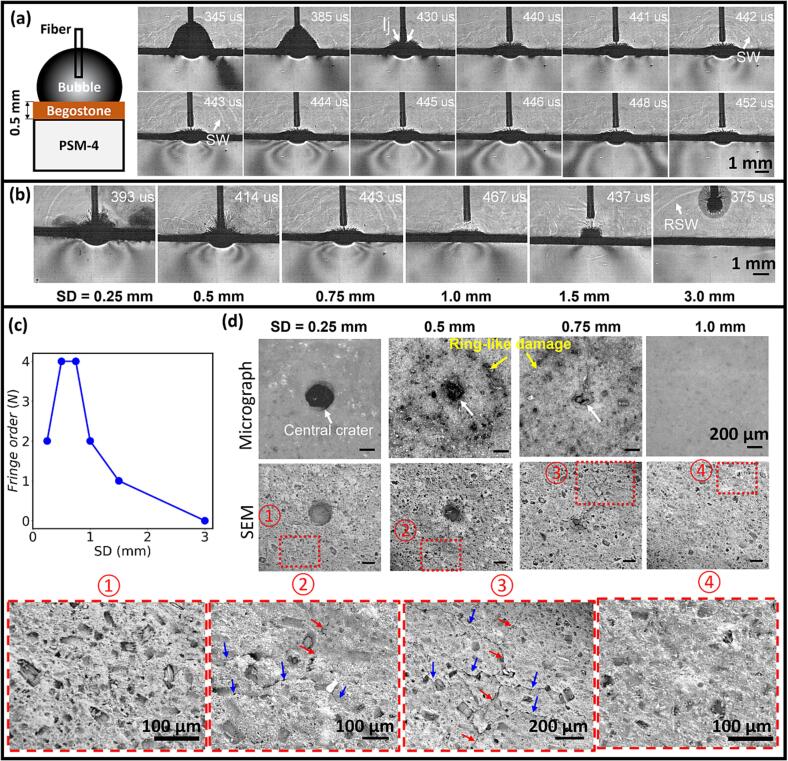
Bubble-stone interaction with resultant stress field and material damage produced by Ho:YAG laser (0.2 J/10 Hz) induced cavitation bubble collapses at different standoff distances (SDs). (a) Dynamic shadowgraph/photoelastic images of the bubble dynamics in the fluid (top half) and the stress field revealed in the PSM-4 photoelastic material (bottom half) at SD = 0.75 mm. A thin layer of hard BegoStone material was glued to the PSM-4 block; “lj” denotes liquid jet generated by the asymmetric collapse of LL-produced bubble, and “SW” denotes shock wave. (b) Snapshots of the photoelastic imaging showing the maximum fringe order produced by the cavitation bubble collapse for different SDs. ‘RSW’ denotes reflected shock wave. (c) Quantitative comparison of the maximum fringe order observed in the PSM-4 material under different SDs. (d) Representative micrograph and SEM images of the BegoStone surface damage after 20 laser pulses. White arrow points to the central crater, yellow arrows highlight the ring-like damage surrounding the central crater. Red and blue arrows indicate micro-fractures in the ring-like damage region along the radial and circumferential directions, respectively. No surface damage was observed at SD ≥ 1 mm. (For interpretation of the references to colour in this figure legend, the reader is referred to the web version of this article.)

To understand the causal relationship between the toroidal bubble collapse and stone damage, we further perform high-speed shadowgraph/photoelastic imaging to capture the transient collapse of LL-generated bubbles at different SDs and the resultant stress fields (revealed by the fringe patterns) produced in the solid boundary. As shown in Fig. 8a (and Supplementary Movie S10), a slab of hard BegoStone (0.5 mm thick) was glued to a PSM-4 block (90 × 8 × 20 mm, *L* × *W* × *H*), which exhibits birefringence and can thus be used qualitatively as a stress sensor in photoelastic imaging [63], [64]. At SD = 0.75 mm the LL-generated bubble collapses asymmetrically with a liquid jet impinging upon the BegoStone surface during 430–440 μs. Subsequently, a pair of fringes are observed in the PSM-4 block, expanding laterally on the solid boundary. At 442 μs, the shock waves emitted in the fluid are first observed, followed by the appearance of maximum fringe order (*N*_max_ = 4) with the highest spatial gradient produced in the PSM-4 material at 443 μs. Importantly, the pair of fringes created at the surface and propagating along the solid boundary suggest the possibility of surface acoustic wave generation [48], [65] by the collapse of the toroidal bubble.

Quantitatively, at SD = 0.5 and 0.75 mm, the primary bubble collapse produces the highest fringe order (*N*_max_ = 4) (see Fig. 8b and c), corresponding presumably to the highest stress generated near the solid boundary. As SD increases from 1.0 to 1.5 mm, *N*_max_ decreases from 2 to 1. At long SD = 3.0 mm, the emission sites of the shock waves produced by the bubble collapse are far away from the solid boundary (see Fig. 4a) and no fringes are observed in the PSM-4 material (Fig. 8b). It should be noted that at short SD = 0.25 and 0.5 mm, the damage crater extends through the BegoStone layer, creating a burn mark in meniscus-shape on the photoelastic material surface right underneath the fiber tip.

After the LL treatment, the common damage features on the BegoStone surface (Fig. 8d) are examined by optical and scanning electron microscopy (SEM). At SD = 0.25–0.75 mm, a central damage crater is produced at the laser irradiation spot, presumably due to photothermal ablation. The diameter of the central crater (0.34 mm) matches closely to the size of the laser spot under SD = 0.25 mm (Fig. 2d). Moreover, this central crater becomes smaller and shallower with increased SD, as observed previously [23]. Most interestingly, at SD = 0.5 and 0.75 mm, there are multiple pitting damages formed along a ring surrounding the central crater. The radii of the ring-like damage are 0.6 and 0.7 mm for SD = 0.5 and 0.75 mm, respectively, which match well with the radii of the toroidal bubble during its primary collapse (see Fig. 7c). Furthermore, the high-magnification SEM images show clearly micro-fractures with a length scale about 100–300 μm in the ring-like damage region, suggesting a possible mechanism for dust generation by the collapse of the toroidal bubbles. In comparison, no appreciable pitting or microcrack formation is observed in the SEM images from the amorphous and porous stone surfaces treated at SD ≥ 1.0 mm.

**Fig. 9:**
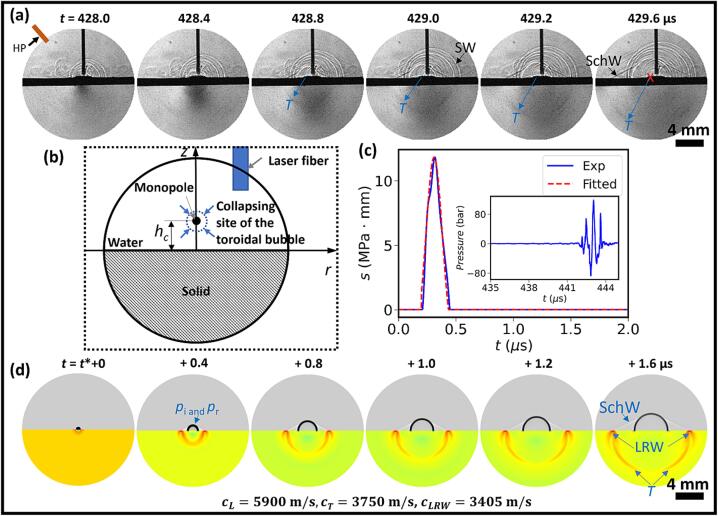
Experimental and simulation results of the shock wave interaction with a solid (quartz) boundary produced by the primary collapse of laser lithotripsy-generated bubbles. (a) Dynamic photoelastic/shadowgraph images of the shock wave emission and transverse (T) or shear wave generation from bubble collapse near the quartz boundary captured at 5 Mfps under a pulse energy of 0.2 J and standoff distance (SD) of 0.5 mm. “HP” denotes the hydrophone, and “SchW” denotes the Schmidt head wave. (b) Schematic diagram of the numerical simulation setup. (c) Pressure waveform measured by a hydrophone in water and the fitted source strength based on the peak pressure profile. (d) Numerical simulations of the transient pressure and elastic waves generated in water and solid, caused by a monopole point source interaction with the solid boundary. Here, “*p_i_* and *p_r_*” denotes the incident and reflected pressure waves in the fluid, “*T*” denotes the transverse wave, and “*LRW*” denotes the leaky Rayleigh wave.

We further repeated the shadowgraph/photoelastic imaging experiment near a quartz boundary at 2 Mfps (Fig. 9a), which revealed the shock wave emitted by the primary bubble collapse propagating in water at a speed of 1820 m/s from 428.4 to 429.6 μs, corresponding to a Mach number (Ma=v/c, *c* is 1480 m/s at 20 °C) of 1.23. Moreover, the shear wave in the solid excited by the shock wave-quartz interaction could also be observed, propagating at a speed of 3780 m/s. From the shock front geometry, the origin of the shock wave emission site (marked as “x” in Fig. 9a) was traced back to about 0.55 mm left of the laser central axis, confirming again that the shock wave was generated by the collapse of the toroidal bubble (see Fig. 5, Fig. 6). Another distinct feature observed is the Schmidt head wave in the fluid, propagating at a characteristic angle of 25.1° from the solid surface normal [65], indicating the excitation of leaky Rayleigh wave (*LRW*) at the water–quartz boundary by the collapse of the toroidal bubble.

Next, we simulated the shock wave emitted from the toroidal bubble collapse by a monopole point source with its maximum strength profile fitted with the acoustic pressure measured in Fig. 9a using a multiphysics fluid–structure interaction model [65] (see Fig. 9(b, c)). The numerical simulations clearly revealed the pressure waves (*p_i_* and *p_r_*) and the Schmidt head wave in the fluid, as well as the transverse (*T*) or shear wave in the solid and the *LRW* characterized by the dual branch (fish-tail) structure propagating along the water–quartz boundary (Fig. 9(d)). The simulation results matched reasonably well with the experimental observations.

**Fig. 10:**
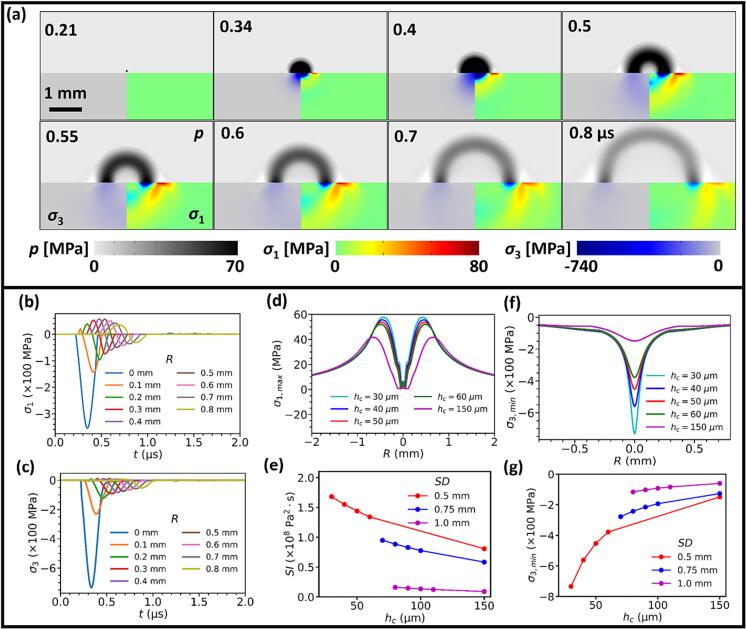
COMSOL simulations of the shock wave emitted by a monopole point source mimicking a toroidal bubble collapsing spot and its interaction with nearby BegoStone boundaries under SD = 0.5 mm and *h*_c_ = 30 μm, unless noted otherwise. (a) The contour plots of pressure in the water and the first (σ_1_) and third principal stress (σ_3_) in the stone. (b) Time history of σ_1_ on the stone boundary. (c) Time history of σ_3_ on the stone boundary. (d) Radial distribution of σ_1, max_ on the stone boundary under different *h*_c_. (e) Variations of the maximum stress integral (SI) produced on the stone boundary vs. *h*_c_ at different SDs, here SI=$\int_{0}^{T}{\left(\sigma_{T}-\sigma_{0}\right)}^{2}dt$, for ($\sigma_{T}>\sigma_{0}$), $\sigma_{0}=7.1$ MPa for hard BegoStone. (f) Radial distribution of σ_3, min_ on the stone boundary under different *h*_c_. (g) Variations of the maximum compression stress vs. *h*_c_ at different SDs.

Finally, we assess the response of the BegoStone material subjected to the transient shock wave loading from the toroidal bubble collapse based on the experimental measurements under SD = 0.5, 0.75 and 1.0 mm (see Fig. 5 and Fig. 7c, also summary in Table S2). As shown in Fig. 10a for SD = 0.5 mm and *h*_c_ = 30 μm, the maximum compressive stress is produced right underneath the pressure source by the initial shock wave-stone interaction and decays rapidly as the resulting *L* and *T* waves propagate into the bulk of the stone material. In contrast, the maximum tensile stress is produced on the stone boundary by the *LRW* [47], which propagates along the fluid–solid interface ahead of the pressure waves in the fluid. The time history of first (maximum tension) and third (maximum compression) principal stresses (σ_1_ and σ_3_) produced on the solid boundary at different lateral radial distances (*R*) are shown in Fig. 10b and c. The simulation results demonstrate that while σ_1_ is unipolar and compressive along the axis of symmetry (*R* = 0 mm), a bipolar pulse profile with a leading tensile stress (positive) followed by a compressive (negative) stress is developed at off-axis locations (|*R|* > 0 mm). The tensile stress amplitude initially increases with *R*, reaching a maximum between 0.4 and 0.5 mm before decaying monotonically thereafter due to the loss of re-radiated acoustic energy into the fluid by the Schmidt head wave [47]. At SD = 0.5 mm (Fig. 10d), a maximum peak tensile stress of 57.8 MPa is produced at *R* = 0.44 mm under *h*_c_ = 30 μm. When the collapse location of the toroidal bubble varies in the range of *h*_c_ = 30–60 μm, the peak tensile stress will only decrease by 10 % while the associated radial location is shifted slightly outward by 0.05 mm. The −6 dB width of the maximum tensile stress is about 0.8 mm. This result suggests that under the shock wave impacts, the *LRW* excited along the fluid–solid interface may cause microcrack formation (see Fig. 8c) near the collapsing sites of the toroidal bubble close to the stone surface. In comparison, σ_3_ is largely unipolar and compressive (negative) at various radial locations on the stone boundary (Fig. 10c). The maximum compression (σ_3, min_ = −740 MPa) is produced at the projected center of the shock wave emission on the stone boundary (i.e., *R* = 0 mm) with −6 dB width of 0.24 mm as the incident acoustic energy is converted partially into the elastic waves in the solid. Considering the microcavities in the porous BegoStone material (see Fig. 8c), compression-induced tensile failure [66] may contribute to the damage underneath the stone surface subjected to the strong shock loadings inflicted by the intensified toroidal bubble collapse. It is worth noting that in the same variation range of *h*_c_ = 30–60 μm, the peak compressive stress will drop significantly by 50 % while the location of the maximum compression remains unchanged at *R* = 0 mm. This finding suggests that the compression-induced tensile failure will be limited to a narrow region directly underneath the shock impact site.

Based on the Tuler-Butcher criterion for brittle material damage under dynamical loading, we calculated the stress integral (SI) associated with the maximum tension produced by the toroidal bubble collapse near the stone boundary. As shown in Fig. 10(e), the value of SI will decrease almost linearly with *h*_c_. Moreover, significantly higher SIs are produced at short SD of 0.5 mm with small *h*_c_ of 30 to 60 μm than long SD of 1.0 mm with large *h*_c_ of 80 to 110 μm, while the corresponding values for SD = 0.75 mm and *h*_c_ of 70 to 100 μm are in between. Similarly, the peak compression is found to decrease significantly with increasing *h*_c_ and SD (Fig. 10g).

## 4 Discussions

Ho:YAG lasers, operating in the near infrared range (λ = 2.08 μm) with a strong absorption in water, have been the gold standard of LL in the past two decades because of their effectiveness in treating kidney stones of all compositions [13]. The shallow optical penetration depth in water (∼0.4 mm) at this wavelength also ensures the safety of Ho:YAG LL when the fiber tip is kept at a few millimeters distance from the tissue surface [9].The conventional theory of LL attributes stone damage primarily to photothermal ablation [19], mediated by cavitation-induced vapor tunnel for laser transmission. In contrast, our recent studies [23] have challenged this paradigm and demonstrated the significant contribution of vapor bubble collapse in stone dusting, which is gaining increasing clinical popularity because of the shortened procedure time with lessened ureteral injury [14], [67]. Nevertheless, the physical mechanism of stone damage produced by bubble collapse is still not well understood, and the intricate laser-fluid-bubble-stone interaction needs to be further elucidated in order to improve the efficacy and safety of LL. Addressing this fundamental topic is especially important in the new era when high power and high frequency lasers are increasingly used for the surgical management of KSD patients [68].

In this study, we perform a comprehensive series of investigation both experimentally and numerically to dissect the complex physical processes in LL. We have demonstrated that under clinically relevant conditions for stone dusting (e.g., *E_p_* = 0.2 J, *F* = 20 Hz, SD = 0.5 mm), most of the irradiated laser energy (≥63 %) will be absorbed by the interposing fluid between the fiber tip and stone surface, while less laser energy (≤37 %) will be delivered to the target stone (Fig. 3). In the interposing fluid, although less than 19.5 % of the *E_p_* is needed to initiate cavitation via superheating (Fig. 3g), most of the absorbed laser energy will be lost in heat conduction (>99.7 %), the rest may be lost in acoustic radiation and through other unknown mechanisms (Fig. 3i). The absorbed energy associated with the bubble inception will be partly converted into the kinetic energy (0.02 %) of the fluid around the bubble and the potential energy accumulated at the maximum bubble expansion (0.7 % for the bubbles shown in Fig. 4a). Moreover, as the treatment progresses, additional incident laser energy may be absorbed and/or scattered in the dust-laden fluid with residual minute-bubbles, further increasing the energy loss in the interposing fluid. In fact, recent thermal measurements have indicated that more than 90 % of the laser energy will be lost through heat generation in the fluid during dusting treatments [69].

In comparison, the laser energy transmitted to the stone are most likely to be scattered (Fig. 3a), and eventually lost in heat conduction through sublethal temperature rise either in the stone below the material’s melting threshold or in the surrounding fluid. The burn-mark around the center crater produced by laser treatment in air (Fig. 2d) clearly suggests the creation of a large temperature gradient dispersing radially outward from the laser beam axis. In contrast, the absence of such burn-mark after laser treatment in water further supports the notation that heat is rapidly removed from the laser irradiated spot on the stone surface, presumably by the shear flow in the surrounding fluid associated with the concomitantly generated bubble expansion, collapse, and resultant microstreaming [70], [71]. Such localized streaming flow may further help to remove the damaged materials from the crater, and thus enhancing the effectiveness of photothermal ablation produced by the ensuing pulses. Based on the simulation results (Fig. 3d and 3e), it is interesting to note that only about 1 % of the *E_p_* is needed to create the damage crater via thermal ablation. Furthermore, such a process will saturate within 200 pulses when the crater depth and surface area have grown sufficiently, leading to the energy flux density at the enlarged crater surface fallen below the damage threshold for thermal ablation [51]. Other factors that may dissipate energy during LL, such as deposition of damaged materials on the crater surface, change of the optical and thermal properties of stone materials at elevated temperatures, and micro-explosion in the water-filled pores inside the stone materials [72], [73] are not considered in our current model and will need to be incorporated in future studies.

Because the peak laser irradiance during stone dusting is on the order of 10^6^ W/cm^2^, which is significantly below the threshold for optical breakdown in water (∼10^8^ W/cm^2^), cavitation in Ho:YAG LL is formed by superheating [16] with a rapid phase transition from liquid to vapor gases, through which significant pressure is created inside the bubble to drive its expansion (Fig. 2h). Compared to ns laser generated bubble through optical breakdown, several unique differences have been observed in the bubble dynamics during Ho:YAG LL (Fig. 4). These distinct features include: 1) elongated expansion along the fiber axis with generation of high vapor content inside the bubble and low energy conversion efficiency in bubble formation and expansion, 2) dampened collapse of the vapor bubble with substantially reduced jetting speed (partially due to interaction with the fiber tip and cone bubble formation) and weaker acoustic emission, and 3) strong rebound after the primary collapse, followed by a substantial secondary collapse near the solid boundary. Nevertheless, when the vapor bubble is created closely to a solid boundary (*γ* < 0.9), the progressively intensified pressure transients at small *γ* is similar to those produced by ns lasers yet at a much lower energy utilization efficiency (Fig. 4e). The difference may be attributed to the significant vapor content produced inside the LL-induced bubbles, coupled with the concomitantly increased compression of the gaseous content during the final stage of the bubble collapse under such conditions [29].

Remarkably, despite that only 0.4–0.7 % of the *E_p_* is converted to *E_B_* at the maximum bubble expansion, the collapse of vapor bubble can still deliver sufficiently strong acoustic pressure transients to multiple spots on the stone boundary to cause surface damage under SD < 1 mm (or *γ* < 0.6). Ultra-high-speed imaging combined with simultaneous hydrophone measurement have revealed that the shock wave emission is not produced by the jet impact, but rather by the non-unison and sequentially intensified collapse of the toroidal bubble outside the photothermal ablation zone (Fig. 5, Fig. 6, Fig. 7). The characteristics of the vapor bubble expansion and collapse vary critically with SD (Fig. 4), with the strongest shock wave emission and pressure impulse produced at the sweet spot SD = 0.5 mm (γ= 0.31) where the ring-like damage outside the central crater is most pronounced (Fig. 2d and e). This ring-like damage is consistent with the circular damage pattern produced on solid boundaries by the collapse of ns laser- or spark-generated bubbles in the similar γ range [32], [74]. Moreover, it is important to note that under such conditions, the ring-like damage will disappear when the collapse of the vapor bubble is significantly altered by the presence of a competing solid boundary closer to the fiber tip than the stone surface [75]. Such a maneuver, however, will not affect the bubble expansion (or the MOSES effect) and therefore, we interpret that the central crater is produced predominantly by the photothermal ablation.

In the extreme case when the fiber tip is in contact with the stone surface (i.e., SD = 0 mm), most of the laser energy will be transmitted to the stone material with minimal absorption in the fluid, leading to the generation of a small bubble and hence markedly reduced cavitation damage [75]. This scenario is distinctly different from ns-laser induced bubble in water through optical breakdown at small γ (<0.3) where violent collapse with strong shock wave emission is generated by the progressively increased compression of a large hemispherical bubble [32], [55]. In comparison, when the fiber tip is sufficiently distant from the stone surface, e.g., SD = 3 mm (γ= 1.86), all the laser energy will be absorbed in the fluid, generating a large bubble with strong pressure transients registered upon its collapse (Fig. 4a and Fig. 5d). However, the resultant acoustic emission will diverge rapidly with increasing distance from the bubble collapsing site due to geometrical spreading. As a result, substantially reduced pressure amplitude will impact on the stone surface, incapable of producing material damage [29].

One of the distinct features in LL-induced cavitation is the gas–liquid interface instability, which leads to asymmetry in the final collapsing process of the toroidal bubble (Fig. 5, Fig. 6, Fig. 7) and resultant damage (Fig. 2d). Raleigh-Taylor instability [76] is responsible for the initial breakup of the toroidal bubble into multiple segments (Fig. 6b) due to the acceleration of the bubble surface by the jet impact. Imperfect fiber tip preparation or alignment with the solid surface will create asymmetry in bubble expansion and collapse, leading to instability in the radial splashing flow and unsynchronized collapse of the bubble torus along its circumferential direction (Fig. 5b and Fig. 7a). Subsequently, the shock waves generated by the collapse of small segments will interact with the remaining large segments on the shrinking bubble torus (Fig. 5b), further creating Richtmyer-Meshkov instability [77]. As a result, the shock wave-bubble interactions [78] (including both the incident and reflected shock waves, as well as the Schmidt head waves shown in Fig. 10a and 10d) and successively boosted collapses are produced at multiple spots, releasing acoustic energies locally in close proximity to the stone surface to cause shallow individual craters and formation of microcracks (Fig. 2d and Fig. 8c). Dissimilar toroidal bubble collapsing dynamics and associated surface damage patterns have also been recently demonstrated in LL under fragmenting mode using a parallel fiber alignment at different SDs and *E_p_* = 0.8 J [79].

It should be noted that although progressive collapse of the toroidal bubble generated by ns-laser at γ = 0.3 was previously observed by Philipp and Lauterborn (see Fig. 11 in [32]), there were subtle differences in the characteristic damage produced on aluminum surfaces. In particular, the damage is epitomized by a deep pit at the final collapsing site of the bubble torus (see Fig. 19c in [32]). Reuter et al. [80] further investigated this phenomenon in detail for γ ≤ 0.2 and provided convincing evidence that a self-intensified collapsing process of the toroidal bubble is responsible for producing the deep-drilled pit on various metal surfaces. At γ ≥ 0.2, it was noted that jet-induced instability would impede self-focusing, yet a less-efficient self-focusing could still produce some damage.

Although surface damage associated with toroidal bubble collapse has been attributed to shock wave emission, the resultant stress field on the solid boundary and fracture propensity have not been assessed previously. In this work, we have adapted a multiphysics model of shock wave interaction at a fluid–solid boundary originally developed for nanopulse lithotripsy (NPL) [47]. Although the source strength of the shock wave emitted by the toroidal bubble collapse is much weaker than the spark-generated shock wave in NPL, the collapsing sites are at least an order of magnitude closer to the stone boundary (*h*_c_ = 30–60 μm in LL vs. *h*_c_ = 500–2000 μm in NPL). As such, the maximum SI produced at SD = 0.5 mm by the toroidal bubble collapse (1.68 × 10^8^ Pa^2^ ∙ s, see Fig. 10e) is on the same order of magnitude for the damage threshold of hard BegoStone materials (6.7 to 8.8 × 10^8^ Pa^2^ ∙ s, [81]). It is also important to note that at SD = 1.0 mm, the maximum SI (0.16 × 10^8^ Pa^2^ ∙ s) will fall an order of magnitude below the material damage threshold, consistent with the negligible cavitation damage observed under such treatment conditions (see Fig. 2d).

Since most kidney stones are brittle materials [82], [83], it is likely the collapse of toroidal bubble in close proximity to the stone surface may lead to microfractures and removal of a thin layer (∼200 µm) of materials due to dynamic fatigue [47], [66]. Such stone damage mechanism and process are distinctly different from the photothermal ablation or microexplosive mechanism in LL, which may lead to fracture lines developed into the bulk of the stone material, resulting in large-sized fragments [84]. In contrast, cavitation damage is characterized by superficial damage and material removal to small fragments over a large surface area, much more desirable for stone dusting in LL.

## 5 Conclusions

In conclusion, we have performed a series of unprecedented experiments and numerical simulations to investigate the complex laser-fluid-bubble-solid interaction during Ho:YAG LL and resultant stone damage. Our results suggest that during stone dusting (e.g., *E_p_* = 0.2 J, *F* = 20 Hz, SD = 0.5 mm), most of the irradiated laser energy (>90 %) will be partitioned eventually into heat generation and temperature rise in the fluid surrounding the fiber tip and near the irradiated stone surface. About 1 % of the laser pulse energy transmitted to the stone surface is consumed for photothermal ablation, and less than 0.5 % is converted into cavitation potential energy at maximum bubble expansion, which will be released subsequently upon bubble collapse to cause to surface erosion. Damage produced by photothermal ablation (i.e., the central crater) is confined locally to the laser irradiation spot, whereas multi foci ring-like damage outside the central crater is produced by cavitation bubble collapse under small SDs of 0.5–0.75 mm (0.3 < *γ* < 0.5). Combining ultra-high-speed (shadowgraph, photoelastic and total internal reflection) imaging and hydrophone measurement, we have uncovered that the cavitation damage is not produced by the jet impact, but rather by the progressively intensified collapse of toroidal bubble in proximity to the solid surface (<100 μm) and resultant shock wave emissions, substantiated by the multiphysics model simulations. Overall, this study elucidates the cavitation damage mechanism in stone dusting during Ho:YAG LL. The fundamental physical insights gained may also benefit other research fields, such as laser-mediated surface cleaning [71], microfluidic applications [85], [86], synthesis of nanomaterials [87], and cavitation damage mitigation in hydraulic machines, such as marine propellers and pipelines [26], [88].

## Declaration of Competing Interest

The authors declare that they have no known competing financial interests or personal relationships that could have appeared to influence the work reported in this paper.
