# Supplementary material for: Shock waves generated by toroidal bubble collapse are imperative for kidney stone dusting during Holmium:YAG laser lithotripsy
Source: Ultrason Sonochem. 2023 Oct 15;101:106649. doi: 10.1016/j.ultsonch.2023.106649 (PMC10623368; doi:10.1016/j.ultsonch.2023.106649)
Supplement: Supplementary data 11 [file mmc11.docx]

**Supplementary Information**

**Shock Waves Generated by Toroidal Bubble Collapse are Imperative for Kidney Stone Dusting during Holmium:YAG Laser Lithotripsy**

Gaoming Xiang, Junqin Chen, Derek Ho, Georgy Sankin, Xuning Zhao, Yanguanchen Liu, Kevin Wang, John Dolbow, Junjie Yao and Pei Zhong

**Supplementary Figure**


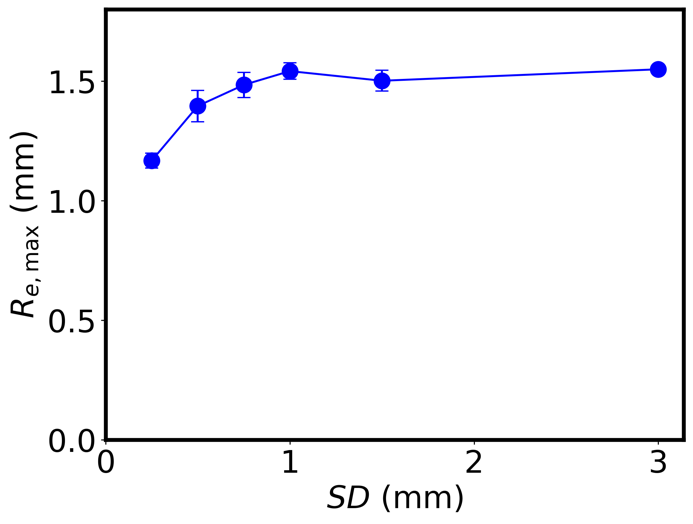


Figure S1. The equivalent radius of the vapor bubble during laser lithotripsy at maximum expansion *R*_e, max_ vs. SD.

**Supplementary Tables**

Table S1. Optical properties of water [1] and soft BegoStone. The optical properties of the soft BegoStone are measured using UV-Vis-NIR Spectrophotometer with Integrating Sphere (UV-3600i with ISR-603 Integrating Sphere Attachment, Shimadzu) with the detailed operation procedure described in [2].

|  | Scattering coefficient, *µ*_s_ (cm^-1^) | Absorption coefficient, *µ*_a_ (cm^-1^) | Refraction index |
| --- | --- | --- | --- |
| Water | 0 | 24 | 1.33 |
| Soft BegoStone | 25.64 | 2.38 | 1.5 |

Table S2. Acoustic and mechanical properties of quartz and soft and hard BegoStone.

|  | *ρ*  (kg/m3) | *c*_P_  (m/s) | *c*_S_  (m/s) | *c_LRW_*  (m/s) | *ν* | *E*  (GPa) | *K*  (GPa) |
| --- | --- | --- | --- | --- | --- | --- | --- |
| Soft BegoStone | 1563 | 3148 | 1813 | 1836 | 0.25 | 12.9 | 8.6 |
| Hard BegoStone | 1995 | 4159 | 2319 | 2187 | 0.27 | 27.4 | 20.2 |
| Quartz | 2200 | 5900 | 3750 | 3405 | 0.17 | 72.0 | 37.0 |

**References**

[1] Fried, N.M., *Recent advances in infrared laser lithotripsy.* Biomedical Optics Express, 2018. **9**(9): p. 4552-4568.

[2] Prahl SA. Everything I think you should know about Inverse Adding-Doubling. Oregon Medical Laser Center, St. Vincent Hospital, 2011. 1344: 1-74.

***Descriptions of the Supplementary Videos***

**Movie S1 (separate file).** Detailed high-speed shadowgraph images captured at 5 million fps from 45 deg view at SD = 0.75 mm, which show the jet impact, toroidal bubble formation and collapse with shock wave emissions. Frames of this video are presented in Fig. 5b.

**Movie S2 (separate file).** Total-internal-reflection (TIR) images captured at 1 million fps during stone dusting (0.2 J and 20 Hz) in Ho:YAG laser lithotripsy, SD = 0.5 mm. Frames of this video are presented in Fig. 6b.

**Movie S3 (separate file).** Total-internal-reflection (TIR) images captured at 1 million fps during stone dusting (0.2 J and 20 Hz) in Ho:YAG laser lithotripsy, SD = 0.75 mm. Frames of this video are presented in Fig. 6b.

**Movie S4 (separate file).** Total-internal-reflection (TIR) images captured at 1 million fps during stone dusting (0.2 J and 20 Hz) in Ho:YAG laser lithotripsy, SD = 1.0 mm. Frames of this video are presented in Fig. 6b.

**Movie S5 (separate file).** Total-internal-reflection (TIR) images captured at 1 million fps during stone dusting (0.2 J and 20 Hz) in Ho:YAG laser lithotripsy, SD = 1.5 mm. Frames of this video are presented in Fig. 6b.

**Movie S6 (separate file).** High-speed shadowgraph images of the final stage of the primary toroidal bubble collapse at SD = 1.5 mm captured at 5 million fps from 45° view angle. Frames of this video are presented in Fig. 6a.

**Movie S7 (separate file).** High-speed shadowgraph images of the final stage of the primary toroidal bubble collapse at SD = 1.0 mm captured at 5 million fps from 45° view angle. Frames of this video are presented in Fig. 7a.

**Movie S8 (separate file).** High-speed shadowgraph images of the final stage of the primary toroidal bubble collapse at SD = 0.75 mm captured at 5 million fps from 45° view angle. Frames of this video are presented in Fig. 7a.

**Movie S9 (separate file).** High-speed shadowgraph images of the final stage of the primary toroidal bubble collapse at SD = 0.5 mm captured at 5 million fps from 45° view angle. Frames of this video are presented in Fig. 7a.

**Movie S10 (separate file).** Dynamic shadowgraph/photoelastic images of the bubble dynamics in the fluid (top half) and the stress field revealed in the PSM-4 photoelastic material (bottom half) captured at 200,000 fps, SD = 0.75 mm. Frames of this video are presented in Fig. 8a.
